# INTEGRATING A FRAILTY INDEX INTO THE CMS HIERARCHICAL CONDITION CATEGORY MODEL FOR PREDICTING HEALTH CARE COSTS

**DOI:** 10.1093/geroni/igae098.1263

**Published:** 2024-12-31

**Authors:** Jieun Jang, Ellen McCarthy, Brianne Olivieri-Mui, Sandra Shi, Chan Mi Park, Stephanie Denise Sison, Dae Hyun Kim

**Affiliations:** Yonsei University Graduate School of Public Health, Seoul, Republic of Korea; Harvard Medical School, Boston, Massachusetts, United States; Northeastern University, Boston, Massachusetts, United States; Harvard Medical School/Hebrew SeniorLife, Boston, Massachusetts, United States; Hebrew SeniorLife, Boston, Massachusetts, United States; University of Massachusetts Chan Medical School, Worcester, Massachusetts, United States; Hebrew SeniorLife, Boston, Massachusetts, United States

## Abstract

The Centers for Medicare and Medicaid Services Hierarchical Condition Category (CMS-HCC) score is a standard risk adjustment tool to predict total Medicare costs. We examined whether a claims-based frailty index (CFI) could enhance the predictive capability of the CMS-HCC score. We utilized electronic health records linked with Medicare data, focusing on patients over 65, including surgical inpatients and outpatients from January 2017 to December 2018. Analyzing 1,855 surgical inpatients using logistic regression, we estimated the likelihood of incurring top 20% Medicare costs for 30 days, 90 days, and 1 year after surgery, comparing 1) the CMS-HCC score alone with 2) the CMS-HCC score combined with the CFI. Similarly, for 58,492 outpatients, we employed generalized estimating equations to estimate the likelihood of being in the top 20% of total Medicare costs for 1 year after an outpatient visit, comparing the same two models. The added value of CFI was assessed using net reclassification improvement (NRI) with 3 risk categories (0 to <10%, 10 to <20%, ≥20%) to compare the two prediction models. Including CFI with CMS-HCC score improved NRI by 7.3% for 30-day costs, 10.1% for 90-day costs, 9.8% for one-year costs in surgical inpatients, and 12.3% for annual costs in outpatients. The added contribution of CFI to CMS-HCC significantly improves the model’s ability to identify patients at high risk of incurring substantial medical costs (top 20%). Accounting for patient frailty may enable healthcare systems to more effectively allocate resources and implement targeted interventions, ultimately reducing healthcare expenditures.

